# Comparison of Exergames Versus Conventional Exercises on the Health Benefits of Older Adults: Systematic Review With Meta-Analysis of Randomized Controlled Trials

**DOI:** 10.2196/42374

**Published:** 2023-06-22

**Authors:** Xi Chen, Lina Wu, Hui Feng, Hongting Ning, Shuang Wu, Mingyue Hu, Dian Jiang, Yifei Chen, Yu Jiang, Xin Liu

**Affiliations:** 1 Xiangya School of Nursing Central South University Changsha China; 2 Xiangya-Oceanwide Health Management Research Institute Central South University Changsha China; 3 National Clinical Research Center for Geriatric Disorders Xiangya Hospital Changsha China; 4 Changsha Xingsha Hospital Changsha China; 5 Department of General Practice 921 Hospital of Joint Logistics Support Force The Chinese People's Liberation Army Changsha China

**Keywords:** exergame, exergaming, older adult, elder, geriatric, gerontology, physical function, mental health, systematic review, meta-analysis, meta-analyses, review methodology, RCT, randomized, cognitive function, depression, QOL, quality of life

## Abstract

**Background:**

Conventional exercises (CEs) can provide health benefits for older adults, but the long-term exercise adherence rate is low. As an emerging, stimulating, and self-motivating strategy, exergames (EGs) are defined as combinations of exercises and games that users carry out through physical actions. They can promote exercise, but the health effects of EGs versus CEs on the physical function and mental health (cognitive function, depression, and quality of life) of older adults remain controversial.

**Objective:**

The aim of the study is to compare the health benefits of EGs versus those of CEs for the physical function and mental health of older adults.

**Methods:**

A comprehensive search was conducted from the earliest available date to February 2023 in the following 6 databases: PubMed, Web of Science, Embase, Cochrane, CINAHL, and PsycINFO. All English-language randomized controlled trials comparing the effects of EGs versus those of CEs on the physical function and mental health of older adults, with nearly same physical activity between the 2 interventions, were included. Risk of bias was independently evaluated by 2 authors using the Cochrane risk of bias in randomized trials tool. Two authors independently extracted data. We followed the *Cochrane Handbook of Systematic Reviews of Interventions* to process and analyze the data for meta-analysis. Standardized mean differences (SMDs) and 95% CIs were used for continuous data, and random models were used for analyses.

**Results:**

We included 12 studies consisting of 919 participants in total. Of these, 10 studies were eventually included in the meta-analysis. The results showed that EGs versus CEs exhibited no significant differences in physical (*P*=.13; *τ*^2^=0.31; *χ*^2^_6_=26.6; *I*^2^=77%; SMD=0.37; 95% CI –0.11 to 0.86) or cognitive function (*P*=.63; *τ*^2^=0.01; *χ*^2^_3_=3.1; *I*^2^=4%; SMD=0.09; 95% CI –0.27 to 0.44) effects.

**Conclusions:**

Our findings indicate no significant difference between EGs and CEs in improving the physical function and cognitive function of older adults. Future studies are required to compare the effects of EGs versus those of CEs on cognitive function according to cognitive status, quantify the “dose-effect” relationship between EGs and health benefits, and evaluate the effects of different types and devices of EGs with regard to the health benefits of older adults.

**Trial Registration:**

PROSPERO CRD42022322734; https://www.crd.york.ac.uk/prospero/display_record.php?RecordID=322734

## Introduction

### Background

With fertility rates declining and life expectancies rising, the global population is aging [1]. The number of adults older than 65 years has tripled over the past 50 years and, by 2050, older adults will make up a quarter of the global population [2-4]. Aging can lead to degenerative changes in the physical and cognitive function of older adults, resulting in impaired daily life functions and reducing the independence of older adults, thereby affecting their mental health and increasing the burden of health care [5]. Physical dysfunction is increasingly common at end of life [6], and the global pooled incidence rate of older adult frailty in communities is 43.4 per 1000 person-years [7]. Mental health problems are also common among older adults in China, and it is reported that 21,129 out of 88,417 older adults (23.6%) have these problems [8].

The physical and mental decline of older adults may eventually have serious social and economic consequences for an aging society [9]. For physical decline, frail older people (97/177, 54.5%) needs more health care services than nonfrail older people (30/987, 2.2%) [10,11], and the median hospitalization cost of frail patients is more than twice that of healthy patients (US $44,408 vs US $18,660) [12]. Furthermore, frail people also require continuous care at 5.82-fold the rate of healthy people after discharge [13]. Regarding mental health, studies predict that worldwide care costs for dementia, which is associated with cognitive decline, will increase to US $2 trillion by 2030 [14]. At the same time, the global economic burden of mental disorders in 2010 was similar to that of cardiovascular diseases, higher than that of cancer, and is expected to more than double by 2030 (US $2.5 trillion vs US $6.1 trillion) [15]. Thus, measures must be taken to promote healthy aging. A proven effective strategy is regular physical exercise [16].

Exercise is defined as “planned, organized and repetitive physical activity” [17]. Several studies have shown that conventional exercises (CEs), such as aerobic, resistance, and combined exercise, can improve the physical function and mental health of older adults [18-23]. However, older adults may not exercise due to lack of access to sports venues (eg, the COVID-19 pandemic), inconvenience, or lack of motivation [24]. In addition, the long-term exercise adherence rate for CEs among older adults seems to be low [25,26]. Therefore, an increasing number of studies have considered possible alternatives to CEs.

Exergames (EGs) are a combination of exercise and gaming that allows people to physically interact with a web-based game scene on a screen [27]. It requires the player’s physical performance, as the technology used in the game system tracks the player’s movements to control those in the game, thus immersing the player [28]. For example, in a Kinect-based EG [29], the game uses infrared light and cameras in the Kinect system to capture and track the player’s movements and creates a full-body 3D web-based map, which is rendered by the screen in front of the player. Participants stand in front of the screen, imitate the actions of digital characters on the screen, and adjust their movements in real time based on instant visual and auditory feedback. It can be implemented in community centers, retirement institutions, long-term care facilities, assisted living, nursing homes, burn centers, hospitals, and homes [30,31].

EGs are an interesting strategy for active aging and good mental health [32]. They have been proven to be acceptable, feasible, safe, enjoyable, stimulating, and self-motivating tools [30,33-35]. EGs can be carried out at home, alone, or in groups, which may make it easier for older adults to participate in exercise [35]. In terms of physical function and mental health, in some studies, it is found that the EGs are better than CEs [36-38]; some have reported that EGs are as effective as CEs [39,40]; yet others have concluded that the effects of CEs are better [41]. In short, there is controversy regarding the effects of EGs and CEs on the physical function and mental health of older adults.

### Research Gap and Aim

While 2 systematic reviews have compared the impacts of EGs and CEs on older adults, the results were inconclusive [42,43]. One compared balance and prevention of falls for EGs versus CEs in healthy older adults, and 20 randomized controlled trials (RCTs) were included [42]. EGs were found to have greater improvements in posture control and dynamic balance than CEs [42]. The other review compared the impact of EGs versus CEs on the cognitive function of older adults; it included 13 studies for systematic review and 11 studies for meta-analysis [43]. The results showed no statistical difference between the EGs and CEs in cognitive function [43]. However, these systematic reviews (1) did not distinguish sedentary video games from EGs; (2) searched only PubMed, Web of Science, and Cochrane databases; (3) included non-RCTs and quasi-experiments; (4) did not consider inconsistencies in exercise content between EGs and CEs; and (5) failed to distinguish the effects of EGs alone from the effects of EGs combined with other interventions, such as CEs.

Therefore, a systematic review should be conducted examining all the evidence, using quantitative analysis to compare the impacts of EGs versus CEs on older adults. Our study aims to compare the health benefits of EG versus CE programs for older adults’ physical function and mental health (cognitive function, depression, and quality of life [QOL]). The comprehensive research results may provide a basis for the choice of rehabilitation strategy for the healthy aging of older adults.

## Methods

The PRISMA (Preferred Reporting Items for Systematic Review and Meta-Analysis) was used to report this review [44].

### Search Strategy

PubMed, Web of Science, Embase, Cochrane, CINAHL, and PsycINFO were searched using subject headings and keywords from their inception up to February 2023. We limited the publication language to English. Search strategies are shown in Multimedia Appendix 1. The references of all included studies were reviewed for further relevant research. If more information about relevant research was needed, we contacted the first author.

### Criteria for This Review

#### Types of Studies

Published peer-reviewed reports of RCTs were included. We considered trials in which randomization was implied with at least 2 intervention arms (ie, EGs and CEs). Quasi-randomized studies were excluded. Abstracts, systematic reviews, case reports, and registered trial reports were also excluded.

#### Participants

Studies focused on older adults, where all participants aged 60 years or older, were included. Studies with a hybrid sample (ie, younger and older adults) and older adults with hemiplegia or other paralysis were excluded.

#### Types of Interventions

Activities carried out under sitting conditions and controlled by handheld devices (ie, sedentary video games) were excluded. There must be a group where the only intervention is EGs not combined with any other intervention, such as CEs or cognitive training.

Studies comparing EGs with CEs (eg, aerobic and endurance training, resistance or strength training, multicomponent training, balance training, high-intensity interval training, Tai Chi, yoga, dance, Otago, physical therapy exercises, ball exercise, and treadmill) were included. The CEs performed precisely the same physical activity as the EGs but did not involve web-based feedback.

#### Types of Outcomes

##### Primary Outcomes

Physical function is defined as the ability to perform and complete objectively measured performance-based tasks that assess cardiovascular fitness, muscle strength, flexibility, mobility, and balance [45]. Physical function was measured by the gait speed test, Berg balance test, sit-to-stand test, or 30-second chair stand test.

##### Secondary Outcomes

Cognitive function is defined as a broad set of thinking abilities that can be measured using performance-based tasks [23]. Cognitive function was measured by the Montreal Cognitive Assessment (MoCA) or Mini-Mental Status Exam (MMSE). If a study used both MoCA and MMSE to measure cognitive function, we extracted only the data measured by MoCA because a previous study has shown that MoCA is a better cognitive function measurement method and can detect cognitive heterogeneity well [46].

Depression was measured by the Geriatric Depression Scale. QOL was measured by SF-36 (36 health survey questionnaire).

### Screening Process and Data Extraction

#### Screening Process

Two reviewers (XC and LW) independently conducted literature screening, first screening titles and abstracts to determine whether the research met the inclusion criteria.

Then the full text was obtained to determine whether the study was eligible for inclusion. If 2 reviewers had disagreements, a third reviewer was to be consulted to decide whether to include it. If disagreements could not be resolved through discussion, we would attempt to contact the corresponding author of the study for clarification.

#### Data Extraction

Two authors extracted data independently. The extracted data included first author, age, sample size (female %), population type, dosage of intervention, types of intervention, types of control, outcome, device, and results for the review objectives. We extracted data presented in figure and graph form when 2 review authors independently obtained the same results. Disagreements were resolved by a third reviewer. If data were missing, we would contact authors.

### Quality Assessment

Methodological quality was assessed independently by 2 authors using the Cochrane risk-of-bias tool [47], which includes the following seven contents items: (1) random sequence generation, (2) allocation concealment, (3) blinding of participants and personnel, (4) incomplete outcome data, (5) selective reporting, and (6) other bias.

### Statistical Analysis

We followed the *Cochrane Handbook of Systematic Reviews of Interventions* to process and analyze data for meta-analysis [47]. The results of EGs and CEs were compared after the intervention. All results are continuous variables. Meta-analysis was performed using Review Manager 5.4 software (Cochrane). Standard mean difference and 95% CIs were used for continuous data. Subgroup analyses based on gender distribution were performed. The results were regarded as statistically significant when *P*<.05 [47]. The heterogeneity test was quantified using the *I*^2^ statistic and the chi-square *P* value. The *I*^2^ statistic was considered low, moderate, or large at 25%, 50%, or 75%, respectively [48]. A chi-square *P* value of .05 or less suggests heterogeneous meta-analyzed studies [47]. The random model was selected because the included studies from different populations were heterogeneous. Publication bias was assessed by examining funnel plots.

## Results

### Selected Studies

**Figure 1:**
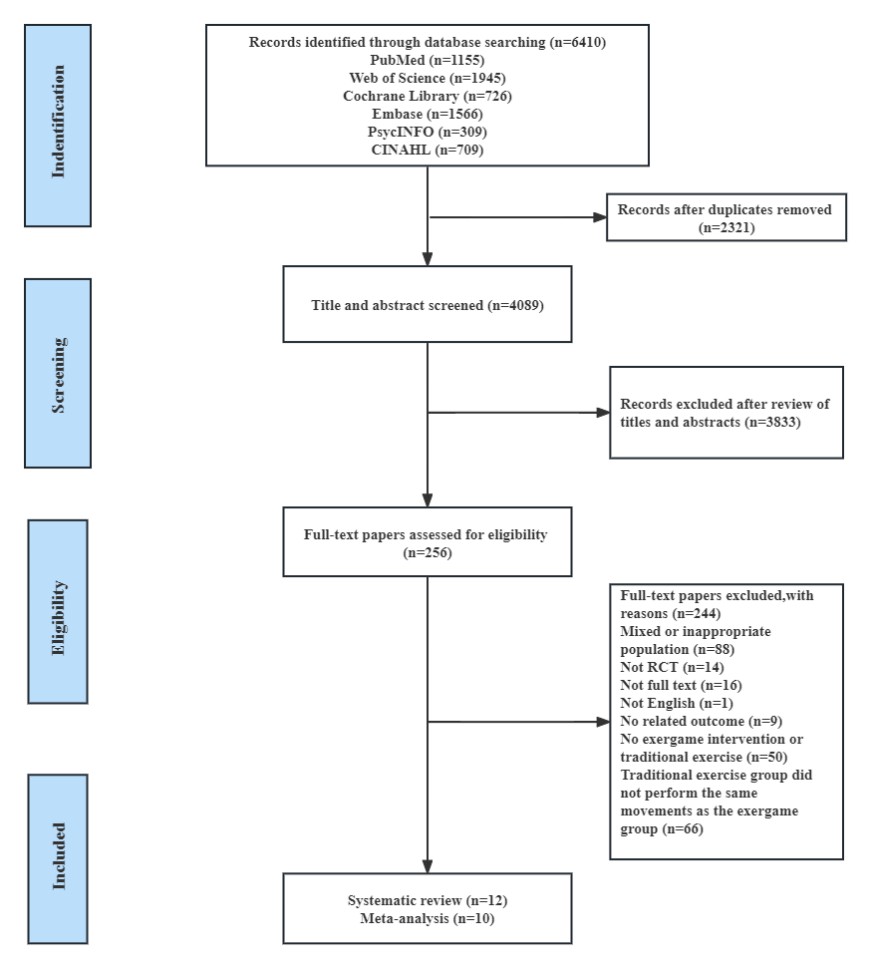
PRISMA (Preferred Reporting Items for Systematic Review and Meta-Analysis) flowchart of the study selection process. RCT: randomized controlled trial.

In total, 6410 potentially relevant studies were identified through database searching. After deduplication, the titles and abstracts of 4089 records were screened by checking the inclusion criteria; 256 studies were further screened by viewing the full texts for eligibility; 12 papers were included for the systematic review, and 10 papers were included for meta-analysis. Figure 1 shows the PRISMA flow diagram for paper selection.

Five studies focused on community-dwelling older adults [38,49-52], 3 studies recruited older adults from care facilities [29,40,53], 1 study was conducted in hospital and home [54], 1 study recruited older adults in a clinical physical rehabilitation unit [55], and 2 studies did not report the research setting [56,57]. Multimedia Appendix 2 [29,38,40,49-57] presents the characteristics of the included studies.

### Characteristics of Participants and Interventions

The 12 included studies had a total sample size of 919 participants, with individual studies ranging from 18 to 282 participants. One study only recruited women [56], 1 study had an equal proportion of female and male participants [55], 6 studies recruited more female participants [29,40,49,52,53,56], and 5 studies recruited more male participants [38,50,51,54,57]. In total, 6 studies focused on older adults with Parkinson disease [38,50,51,54,55,57], 1 on older adults with mild cognitive impairment [49], and 1 on frailty [29]; 4 investigated older adults without special conditions [40,52,53,56].

A 2-arm design was used in 11 studies, including an intervention arm and a control arm [29,38,40,50-57]; 1 study used a 3-arm design, including 2 intervention arms and a control arm [49]. The intervention duration ranged from 5 to 12 weeks, and the most widely used duration was 6 weeks. The frequency was 2 or 3 times per week, and the length of each session was 30 to 90 minutes. The older people in 2 studies only participated in a single session of exercise training [52,53]. In total, 4 studies used Nintendo Wii for the intervention [53,54,56,57], 5 studies used Kinect for the intervention [29,38,49-51], 1 study used Tymo for the intervention [55], 1 study used GRAIL for the intervention [52], and 1 study did not report the intervention device [40].

Among the control group exercises, 1 study consisted of squats, postural displacements, dance, and sports (volleyball and boxing) [53]; 1 included resistance exercise, aerobic exercise, Tai Chi, and balance exercises [29]; 1 included global exercises and balance exercises [57]; 1 used Tai Chi [49]; 1 included task-oriented exercise, walking, stretching, balance training, flexibility exercises, and coordination exercises [55]; 1 included passive range of motion for lower extremities, active free exercises, stretching exercises, strength training, resisted exercise, and balance training [54]; 2 included strength exercises and core training [40,56]; and 4 studies used treadmill training in the control group [38,50-52].

### Meta-Analysis Results

Due to the lack of original data, we did not perform a meta-analysis of 2 papers [38,50].

#### Primary Outcome

**Figure 2:**
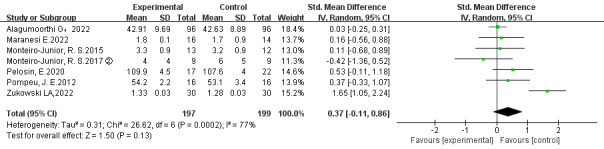
Meta-analysis of the effect of exergames versus conventional exercises on physical function [51-57].

Seven studies reported physical function according to the 30-second chair stand test, gait assessment (GaitRite, CIR Systems, United States), the sit-to-stand test, or the Berg balance test [40,51,52,54-57]. There was no significant difference between EGs and CEs (*P*=.13; *τ*^2^=0.31; *χ*^2^_6_=26.6; *I*^2^=77%; standardized mean difference [SMD]=0.37; 95% CI –0.11 to 0.86), more details in Figure 2.

#### Secondary Outcome

##### Cognitive Function

**Figure 3:**
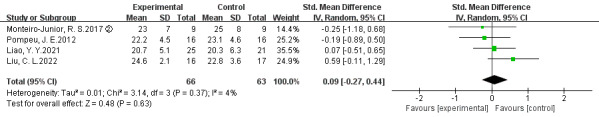
Meta-analysis of the effect of exergames versus conventional exercises on cognitive function [29,49,53,57].

Four studies reported cognitive function according to the MoCA or MMSE [29,40,49,57]. No significant difference was observed between EGs and CEs in MoCA or MMSE (*P*=.63; *τ*^2^=0.01; *χ*^2^_3_=3.1; *I*^2^=4%; SMD=0.09; 95% CI –0.27 to 0.44), more details in Figure 3.

##### Depression

A single study reported depression and found no significant difference between EGs and CEs [40].

##### Quality of Life

Only 1 study reported QOL [38]. The results show that the SF-36 scores of the EG group improved more than those of the CE group.

#### Subgroup Analysis

**Figure 4:**
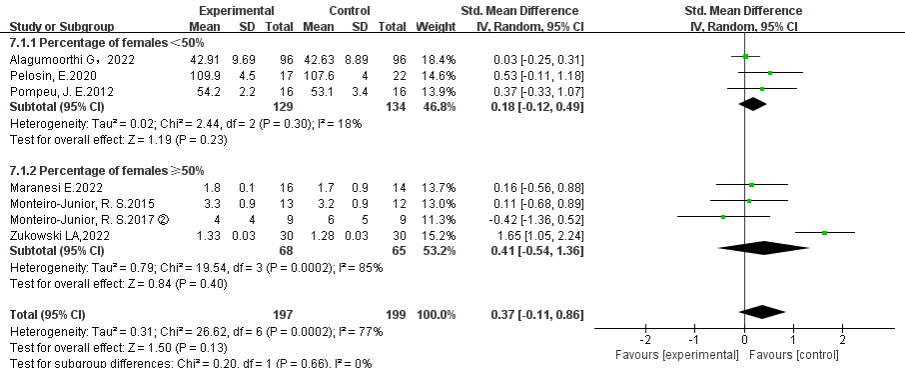
Subgroup analysis of the effects of exergames versus conventional exercises on physical function [51-57].

**Figure 5:**
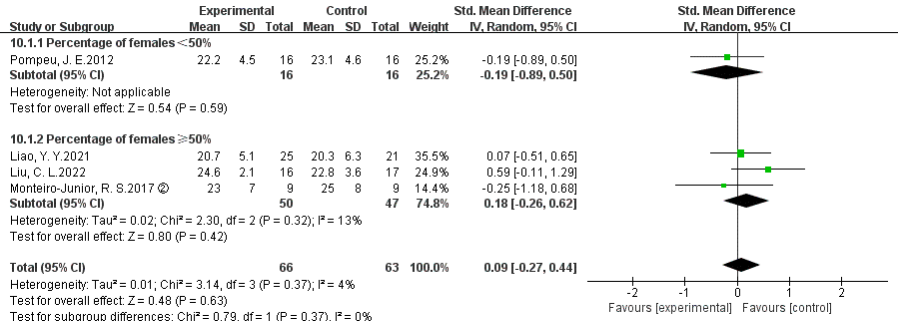
Subgroup analysis of the effects of exergames versus conventional exercises on cognitive function [29,49,53,57].

To further compare the effects of EGs versus CEs on physical and cognitive functions in different gender distributions, the results of subgroup analysis are shown in Figures 4 and 5. No significant difference was observed in physical function between EGs and CEs when the percentage of females <50% (*P*=.23; *τ*^2^=0.02; *χ*^2^_2_=2.4; *I*^2^=18%; SMD=0.18; 95% CI –0.12 to 0.49) and when the percentage of females ≥50% (*P*=.40; *τ*^2^=0.79; *χ*^2^_3_=19.5; *I*^2^=85%; SMD=0.41; 95% CI –0.54 to 1.36). No significant difference was observed in cognitive function between EGs and CEs when the percentage of females <50% (*P*=.59) and when the percentage of females ≥50% (*P*=.42; *τ*^2^=0.02; *χ*^2^_2_=2.3; *I*^2^=13%; SMD=0.18; 95% CI –0.26 to 0.62).

### Publication Bias

**Figure 6:**
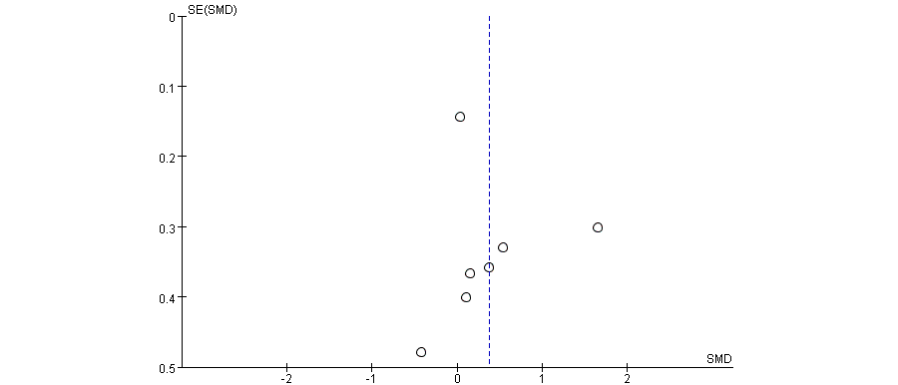
Funnel plot for publication bias assessment. SMD: standardized mean difference.

The funnel plot did not show a clear funnel shape in physical function (Figure 6). The reason may be that some small studies with negative results may not favor the EG intervention.

### Quality Assessment

**Figure 7:**
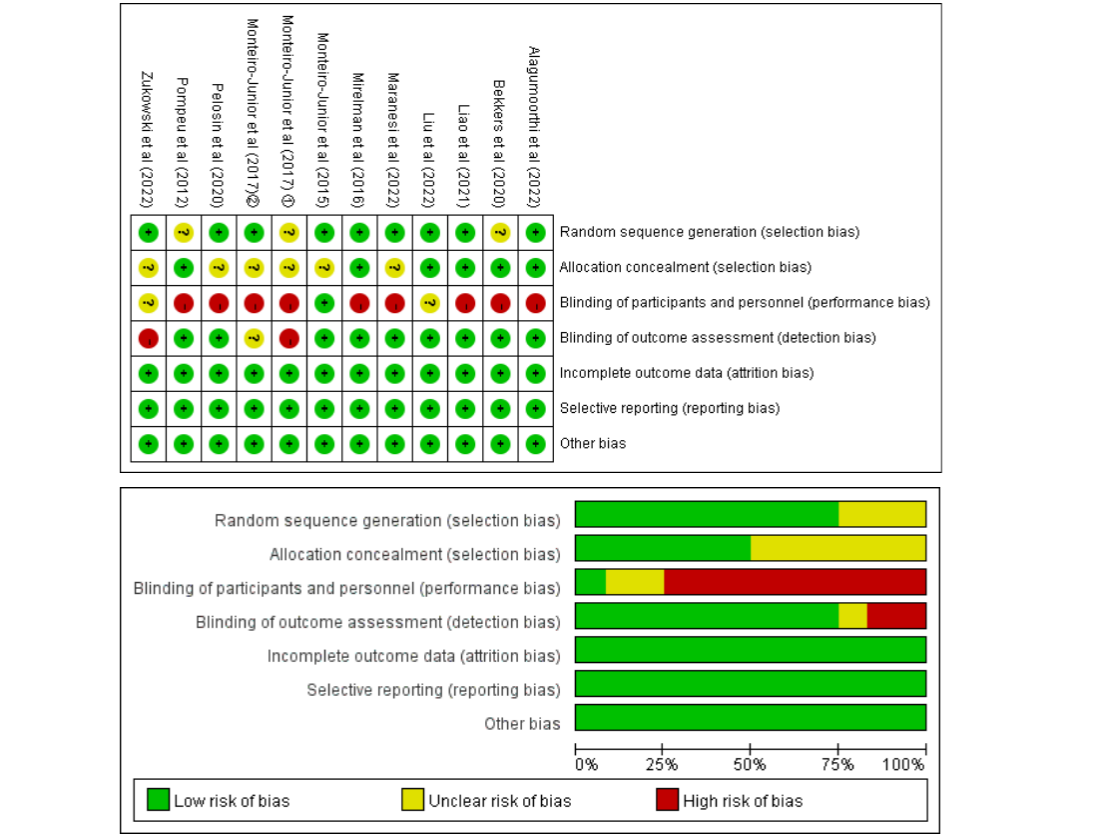
Analysis of the risk of bias in accordance with the Cochrane risk-of-bias tool [29,38,40,49-57].

Figure 7 shows the results of the methodological quality assessment. The quality of the included studies was found to be acceptable. Regarding the risk-of-bias assessment [47], we found that 9 studies showed a high risk of performance bias [29,38,40,50,51,53,57], while 2 studies had unclear bias risk [49,56]. In total, 11 studies used a single-blind protocol [29,38,40,49-55,57], and 1 used a double-blind protocol [56]. Due to the EGs and CEs, it was not possible to blind patients or study personnel to the group allocation. High risk studies may overestimate the effect of EGs compared to CEs.

## Discussion

### Principal Findings

By ensuring that EG and CE groups in the reviewed studies performed the same physical activity, this study is the first to compare the health benefits of EGs and CEs for older adults. We observed that EGs show potential as a novel approach for enhancing physical and cognitive function in older adults. The results of the meta-analysis show no significant difference in physical function or cognitive function between EGs and CEs after intervention. This means that EGs may replace CEs in these aspects. Our findings provide evidence of the beneficial effects of EGs, which may offer a promising strategy for promoting healthy aging in older adults. Given that EGs are an innovative, fun, and relatively safe form of exercise [58], this review presents timely evidence that suggests EGs could be a valuable tool for health professionals, such as physical therapists and occupational therapists.

The results show no significant difference in cognitive function between EGs and CEs. This supports the findings of previous studies [49,59,60]. One RCT with older individuals with dementia showed no significant difference in executive function, episodic memory, or working memory between EGs and aerobic training groups [60]. The other RCT suggested that the Kinect adventures training group and conventional physical therapy group had no significant difference in cognitive function after intervention, and both had positive effects on cognitive function in older adults [59]. In addition, EGs are inexpensive [61], safe [30,62], and easy to use [63], and healthy older adults living in the community can use them without supervision [64,65]. Therefore, the application of EGs in the cognitive rehabilitation of older adults should be promoted, especially for those with mild cognitive impairment or dementia.

However, our findings are inconsistent with a previously published systematic review and meta-analysis that compared the effects of EGs versus CEs on cognitive skills [43]. Based on that study, EGs seem to be more effective than conventional physical training for global cognitive performance. A possible reason is the potential ceiling effect [66]. The previous systematic review and meta-analysis included more patients with mild cognitive impairment or dementia, while our study included more older adults with normal cognitive function. Therefore, even before the intervention, the cognitive function of older adults in our study was quite good, which may make the improvement of cognitive function following EGs and CEs not obvious, resulting in a small difference in the improvement of cognitive function between the 2 groups. Thus, it is suggested that subsequent studies compare the effects of EGs and CEs on cognitive function according to the classification of the cognitive status of older adults.

Furthermore, EGs and CEs showed no significant difference in physical function. This is in accordance with previous findings [67,68]. This may be related to the impact of EGs on heart rate and energy expenditure similar to CEs [69]. Due to a lack of studies, we cannot analyze the impact of potential moderators (intervention time, intensity, and type of EG) on physical function. First, a subgroup analysis of the relationship between intervention time and effect shows that a weekly intervention affects physical function [42]. In that regard, there was no consensus on the duration of weekly interventions in the studies we included. Second, the intensity of EGs must be at least moderate to achieve the effect [69], while the included studies rarely measured the intensity of EGs. Finally, the types and devices of EGs will also affect the intervention effect. It is suggested that future studies explore the impact of different types and devices of EGs on the physical function of older adults. Subsequent studies should quantify the “dose-effect” relationship between EGs and health benefits in older adults, derive optimal intervention doses for EGs (eg, program period, weekly intervention duration, frequency, and intensity), and determine how different types and devices of EGs affect physical function in older adults.

Since gender distribution was very different among the studies, we performed subgroup analysis based on gender but found no difference in physical or cognitive function between groups at different distributions.

Only 1 study compared EGs versus CEs for depression, and only 1 paper compared the effects of EGs and CEs on QOL. The number of such studies is too small to conduct a meta-analysis. Therefore, future research needs to focus more on older adults’ mental health and further explore and compare the effects of EGs versus CEs on depression and QOL.

According to the literature review, in addition to physical function and mental health, the included studies also compared adherence, motivation, cost-effectiveness, fall rates, accessibility, enjoyment, and attractiveness. One study showed no significant difference in adherence between EGs and CEs [38]; one study showed that the adherence of EGs was significantly higher than that of CEs [56]; and another study showed that EGs can improve the motivation and adherence of older adults in the long-term rehabilitation process [57]. One study found that EGs increased participants’ motivation to do more repetitive movements with minimal or no instruction [54]; another study showed that the presence of motivating stimuli and the novelty aspect of EGs can be particularly important in patients with Parkinson disease who have reduced motivation [57]. One study reported that the extra cost of EGs is minimal compared to CEs (the costs of the computer, screen, safety harness, and platform are relatively low for medium-income countries) [38]. Three studies showed that the fall rate of older adults in the EG group was significantly lower than that in the CE group after the training [38,50,54]. Two studies reported that the advantages of accessibility, enjoyment, and attractiveness of EGs for older adults can further enhance the training effect of CEs [29,49]. However, the number of studies was small enough that a meta-analysis could not be done. We recommend that future research compares these outcomes between EGs and CEs because this information would be invaluable to establishing the added value of EGs versus CEs.

It is worth noting that while EGs may replace CEs in improving physical and cognitive function, it should be considered that not everyone is interested in EGs and not all EGs are suitable for older adults. A recent systematic review and qualitative meta-synthesis conducted by our group explored older adults’ experiences of implementing exergaming programs [70]. We found that a small number of older adults were not interested in EGs, which may be due to age- or health-related factors (eg, vision, hearing, motor skills, or cognitive impairment). At the same time, most older adults have no experience with EGs and worry about whether they can understand and play such games correctly. In addition, people in East Asian countries (such as China, Japan, and South Korea) are more likely to feel embarrassed when using EGs because they feel uncomfortable being observed or judged by others. Finally, most existing EGs are not fully suitable for older adults due to a lack of flexibility and adaptability. However, most of these obstacles can be overcome, for example, by designing older people–friendly EGs for different target groups and giving older adults enough time to train and familiarize themselves with EGs.

This paper has limitations. Since we only included English-language studies, information deviations may occur. As exergaming is a fairly new field, there are few papers on this topic, and the number of studies included (and the total sample size) was not very large; thus, the results should be interpreted with caution. The overall methodological quality of the included studies ranged from medium to excellent, so our findings need to be interpreted with caution. Due to the heterogeneity of intervention types, intervention settings, intervention objects, and measurement tools for some outcome indicators, we were unable to conduct a comprehensive meta-analysis. The existence of publication bias resulted in heterogeneity (eg, differences in the intervention protocol) among the included studies, which reduced the quality of evidence. Finally, it must be acknowledged that the conclusions of this systematic review and meta-analysis may have been influenced by the professional backgrounds of the authors.

### Conclusions

Our findings suggest that EGs are a novel and effective strategy for improving physical and cognitive function in older adults. There is no significant difference between EGs and CEs in improving the physical function and cognitive function of older adults, and EGs may replace CEs in these aspects. Our results confirm the effectiveness of EGs in rehabilitation programs for older adults and indicate that EGs may be a novel and feasible alternative to CEs. Future studies should compare the effects of EGs and CEs on cognitive function according to classification of the cognitive status of older adults. Subsequent studies should also quantify the “dose-effect” relationship between EGs and health benefits in older adults, derive optimal intervention doses for EGs, and explore the effects of different types and devices of EGs on physical function in older adults. More high-quality studies with more accurate outcome indicators are needed to further explore and compare the health benefits of EGs versus CEs.

## Appendix

Multimedia Appendix 1 Literature retrieval strategy for the PubMed database.

Multimedia Appendix 2 Characteristics of studies included in this review.

## Abbreviations

CE: conventional exercise
EG: exergame
MMSE: Mini-Mental Status Exam
MoCA: Montreal Cognitive Assessment
PRISMA: Preferred Reporting Items for Systematic Reviews and Meta-Analyses
QOL: quality of life
RCT: randomized controlled trial
SF-36: 36 health survey questionnaire
SMD: standardized mean difference
