# Supplementary material for: Comparison of Exergames Versus Conventional Exercises on the Health Benefits of Older Adults: Systematic Review With Meta-Analysis of Randomized Controlled Trials
Source: JMIR Serious Games. 2023 Jun 22;11:e42374. doi: 10.2196/42374 (PMC10337432; doi:10.2196/42374)
Supplement: Multimedia Appendix 1 [file games_v11i1e42374_app1.docx]

Multimedia Appendix 1

Search strategy.

| PubMed | Searches | Results |
| --- | --- | --- |
| #1 | "Exergaming"[MeSH Terms] | 104 |
| #2 | "Virtual Reality"[ MeSH Terms] | 5221 |
| #3 | "Video Games"[MeSH Terms] | 7108 |
| #4 | exergam*[Title/Abstract] | 1175 |
| #5 | virtual reality exercis*[Title/Abstract] | 85 |
| #6 | "video gam*"[Title/Abstract] | 4886 |
| #7 | "active video gam*"[Title/Abstract] | 359 |
| #8 | "computer gam*"[Title/Abstract] | 1565 |
| #9 | serious gam*[Title/Abstract] | 1389 |
| #10 | Wii*[Title/Abstract] | 1033 |
| #11 | Kinect*[Title/Abstract] | 1621 |
| #12 | #1 OR #2 OR #3 OR #4 OR #5 OR #6 OR #7 OR #8 OR #9 OR #10 OR #11 | 18225 |
| #13 | "Aged"[MeSH Terms] | 3433004 |
| #14 | "Aging"[MeSH Terms] | 288980 |
| #15 | "elder*"[Title/Abstract] | 302926 |
| #16 | "old*"[Title/Abstract] | 1226948 |
| #17 | "senior*"[Title/Abstract] | 50096 |
| #18 | #13 OR #14 OR #15 OR #16 OR #17 | 4709237 |
| #19 | "Randomized Controlled Trial" [Publication Type] | 586876 |
| #20 | "Controlled Clinical Trial" [Publication Type] | 677144 |
| #21 | Randomized[Title/Abstract] | 644311 |
| #22 | placebo[Title/Abstract] | 241986 |
| #23 | "Drug Therapy"[MeSH Terms] | 1494222 |
| #24 | randomly[Title/Abstract] | 401676 |
| #25 | trial[Title/Abstract] | 745980 |
| #26 | groups[Title/Abstract] | 2501407 |
| #27 | #19 OR #20 OR #21 OR #22 OR #23 OR #24 OR #25 OR #26 | 4786167 |
| #28 | "Animals"[MeSH Terms] NOT "Humans"[MeSH Terms] | 5086858 |
| #29 | #27 NOT #28 | 4057433 |
| #30 | #12 AND #18 AND #29 | 1177 |
| #31 | #30 AND (English[Filter])) | 1155 |
