# Supplementary material for: Comparison of Exergames Versus Conventional Exercises on the Health Benefits of Older Adults: Systematic Review With Meta-Analysis of Randomized Controlled Trials
Source: JMIR Serious Games. 2023 Jun 22;11:e42374. doi: 10.2196/42374 (PMC10337432; doi:10.2196/42374)
Supplement: Multimedia Appendix 2 [file games_v11i1e42374_app2.docx]

Multimedia Appendix 2

Study characteristics.

| Study | Sample size (%female) | Age  (years) | Population type | Dosage of intervention | Device | Types of intervention | Types of control | Outcome | Results |
| --- | --- | --- | --- | --- | --- | --- | --- | --- | --- |
| Monteiro-Junior, R. S.2017[53] | 19(84.21) | I:  86 ± 7.0 C:  86 ± 5.0 | long-term care | 30-45min，older adults only participated in a single session of exercise training | Nintendo Wii | Exercises by WG were selected from the two virtual packs of the Nintendo Wii (Wii Fit Plus and EA Sports Active) as follows: Rowing Squat, Penguim Slide, Basic Run Plus, Bump and Set, Heavy Bag and Dance Basic 1. Such games simulate activities as squat, postural displacements, dance and sports (volleyball and boxing) | Control group performed exactly the same movements as the WG. However, this group has not received virtual feedback. | Cognitive function | We found no statistically  significant differences between the two groups. |
| Liao, Y. Y.2021[29] | 46(67.39) | I:  79.6±9.0 C:  83.8±5.1 | daycare centres for the  elderly | 60min,3/wk,12 wk | Kinect | 20 min each of resistance exercise, aerobic exercise, and Tai Chi and balance exercises during exergaming. | The combined physical exercise included 20 min each of resistance exercise, aerobic exercise, and balance exercise | Cognitive function | The group×time interaction indicated that EXER training significantly enhanced global cognition more than CPE training. Moreover, only the EXER group showed significant improvements in verbal and working memory after the intervention. |
| Pompeu, J.E. 2012[57] | 32(46.88) | I:  68.6±8.0 C:  66.2±8.3 | _ | 60min,2/wk,  7 wk | Nintendo Wii | Experimental group received Wii-based motor and cognitive training(balance exercises) and global exercises. | global exercises，balance exercises | Physical function, Cognitive function | There was no difference between the control group and the experimental group before training,  after training or 60 days after training. |
| Liu, C. L.2022[49] | 50(70.00) | Control group: 73.4±6.5 Tai Chi Group: 73.2 ± 6.3  Exergaming-Based Tai Chi group: 74.6 ± 6.1 | community-living | 50min,3/wk,  12wk | Kinect | Exergaming-based TaiChi group participants performed 50 min of TC training during exergaming. | Tai Chi | Physical function, Cognitive function | There were no significant differences between the EXER-TC and TC groups |
| Monteiro-Junior, R. S.2015[56] | 30(100.00) | 68 ± 4.0 | _ | 90min,3/wk,  8wk | Nintendo Wii | This group received the same intervention that control exercise group plus 30 minutes of virtual physical training  (eight exercises) using Nintendo Wii-motion and Wii Balance Board | Control exercise group did strength exercises and core training. | Physical function | There was significant  difference within groups. |
| Monteiro-Junior, R. S.2017[40] | 18(66.67) | I:  85±8.0 C:  86±5.0 | long-term care | 30-45min, 2/wk,  6-8wk | - | This group received the same intervention that control exercise group plus 30 minutes of virtual physical training  (eight exercises) using Nintendo Wii-motion and Wii Balance Board | strength exercises, core training | Physical function, Cognitive function, Depression | ES showed improved outcomes in GPhysEx compared to ACG in  global cognition, executive functions  and short-term memory. 8ftUG was significantly different between  groups showing improved mobility for GPhysEx compared with ACG. |
| Mirelman, A.2016[38] | 282(35.46) | I:  74.2±6.9 C:  73.3±6.4 | community-living | 45min,3/wk,6 wk | Kinect | Training was similar between arms, except for the computerised simulation component for those subjects who were assigned to treadmill training plus virtual reality. | treadmill training | Physical function,  Cognitive function, QOL | Other outcomes (gait  speed variability, leading foot clearance, SPPB balance,  SPPB gait, SF-36 physical total, and SF-36 mental total)  improved more in the treadmill training plus VR group than in the treadmill training group. CF outcomes  improved similarly in both training groups. |
| Bekkers, E.2020[50] | 121(38.84) | I:  71.06±6.3 C:  70.86±6.0 | community-living | 45min,3/wk,  6 wk | Kinect | Training was similar between arms, except for the computerised simulation component for those subjects who were assigned to treadmill training plus virtual reality. | treadmill training | Physical function, Cognitive function | A significant effect of time was found for executive function  (TMT-B scores), indicating that both groups benefited equally from both training modes. |
| Pelosin, E.2020[51] | 39(6.67) | I:  73.2 ± 3.6  C:  71.9 ± 4.1 | community-living | 45min,3/wk,  6 wk | Kinect | Treadmill training with non-immersive virtual reality intervention arm walked on a treadmill while reacting to a virtual environment that included real-life challenges consisting of obstacles, pathways, and distracters. | treadmill training | Physical function | Participants randomized in TT+VR training showed significant improvements of gait kinematics in both the planning and the crossing phases. |
| Maranesi,  E.2022[55] | 15(50.00) | I:  72.7±6.3  C:  75.5±5.4 | Clinical Unit of Physical Rehabilitation | 50min,2/wk,5 wk | Tymo® | The techno-logical intervention group carried out 30 min of traditional therapy and 20 min of treatment  with the Tymo® system (a wireless platform that provides non-immersive virtual reality exergames). | Traditional therapy: breathing and relaxation; task-oriented exercise; walking with cues; stretching; static and dynamic balance training; flexibility exercises; unilateral and contralateral coordination exercises performed in bed and standing involving the 4 limbs. | Physical function | Results suggest how non-immersive virtual reality exergaming technology offers the  opportunity to effectively train cognitive and physical domains at the same time. |
| Zukowski,LA,2022[52] | 43(71.67) | I:  71.2±6.5  C:  72.0±7.7 | community-living | 30min, older adults only participated in a single session of exercise training | GRAIL | The single VRTT sessions were completed on the GRAIL (Motek Medical, Amsterdam,  The Netherlands), a semi-immersive virtual reality system with an instrumented dual-belt treadmill positioned within and integrated with a 180-degree curved projection screen. Participants  completed two versions, an easier and a harder version, of three different exercise games | Treadmill training. It also completed on the GRAIL and required participants to perform the same three exercise games, but with the video projection and audio sounds turned off. | Physical function | The results indicate that both VRTT and CTT may acutely improve gait and cognition.  Therefore, older adults will likely benefit from participating in either type of exercise. The  study also provides evidence that baseline cognition can impact training effects on DTE on cognition. |
| Alagumoorthi,G,2022[54] | 78（40.625） | I:  69.7±10.0  C:  68.5±9.8 | Hospital and home | 60-70min，3/wk,12 wk | Nintendo Wii | 30 to 40 min of Wii-sport (They were cate-  gorised into games that facilitate ankle & hip movements thus induce fixed support strategies, games that facilitate stepping  during turning, getting up & walking thus induce change-in-support strategies) +30 to 40 min of traditional balance training (exercise  for developing fixed support strategies and  change-in-support strategies) | 30 min of conventional physiotherapy(pass  -ive range of motion for lower extremities, active free exercises and  passive sustain stretching exercises, functional strength training,and resisted exercise) +30 to 40 min of traditional balance training (exercise for developing fixed support strategies and change-in-support strategies) | Physical function | A 12 weeks exercise training using the Wii sports-based strategy decreases the number of  fallers, fall rate, measures of risk of falling but did not alter the quality of life in adults with idiopathic  Parkinson’s disease. |

I, intervention group; C, control group; WG: Wii group; wk, week; EXER, Kinect based exergaming; CPE, combined physical exercise; TC, Tai Chi; EXER-TC, exergaming-based TC group; PhysEx, virtual reality based physical exercise with EGs; ACG, active control group; ES, effect size; VR, virtual reality; SPPB, Short Physical Performance Battery; TMTB, Trail Making Test B.
